# Apolipoprotein A-IV involves in glucose and lipid metabolism of rat

**DOI:** 10.1186/s12986-019-0367-2

**Published:** 2019-07-02

**Authors:** Zhenguo Wang, Lu Wang, Zhuzhen Zhang, Li Feng, Xue Song, Jiarui Wu

**Affiliations:** 1 Key Laboratory of Systems Biology, CAS Center for Excellence in Molecular Cell Science, Shanghai Institute of Biochemistry and Cell Biology, Chinese Academy of Sciences, University of Chinese Academy of Sciences, 320 Yueyang Road, Shanghai, 200031 China; 2 School of Life Science and Technology, ShanghaiTech University, 100 Haike Road, Shanghai, 201210 China

**Keywords:** ApoA-IV knockout rats, Glycolysis, Gluconeogenesis, *de novo* lipogenesis

## Abstract

**Background:**

Apolipoprotein A-IV (ApoA-IV) exists in relatively high levels in the circulation systems of animals, but its roles are not fully elucidated. It is known that the *Apoa4* gene resides in the cluster *Apoa1*/*Apoc3*/*Apoa4*. Because of a short intergenic sequence between *Apoc3* and *Apoa4*, a previous ApoA-IV knockout mouse model by gene targeting had an accompanying deficiency in ApoC-III expression, which limited its application in investigating the precise roles of ApoA-IV. To solve this problem, we created a specific knockout of ApoA-IV in Sprague-Dawlay rats by TALEN approach.

**Methods:**

Age-matched knockout rats and their wild-type littermate controls maintained on a standard rodent diet were studied and blood metabolic parameters were measured. Glucose, insulin, olive oil, and intralipid tolerance tests were performed to study the glucose and lipid metabolism of rats. Quantitative real-time PCR and RNA-seq analysis in liver and inguinal white adipose tissue (iWAT) of rats at three ages (18 weeks, 45 weeks and 90 weeks) were performed to identify the genes altered by ApoA-IV knockout.

**Results:**

ApoA-IV knockout rats were apparently normal and fertile, but exhibited improved glucose clearance when challenged with glucose tolerance test. In addition, fasting-induced hepatic steatosis was more pronounced in ApoA-IV knockout rats. Further analysis identified that a set of hepatic genes involved in glycolysis, gluconeogenesis and *de novo* lipogenesis were altered in the absence of ApoA-IV, which induced enhanced glycolysis, attenuated gluconeogenesis and elevated *de novo* lipogenesis. And the RNA-seq results also confirmed that almost all the genes mentioned in the phenotyping section were highly consistent throughout the three studied ages.

**Conclusions:**

ApoA-IV functions in an age-independent manner in the modulation of glucose and lipid metabolism of rats, and may serve as a potential linker between hepatic glucose and lipid metabolism.

**Electronic supplementary material:**

The online version of this article (10.1186/s12986-019-0367-2) contains supplementary material, which is available to authorized users.

## Background

Apolipoprotein A-IV (ApoA-IV), first discovered in rat plasma in the 1970s [1], is a 46 kD glycoprotein with relatively high concentrations in plasma (15–37 mg/dL) [2]. It is expressed predominantly in the mammalian small intestine (with highest expression in the duodenum), and also expressed in rodent liver at lower levels. Sensing lipid absorption in enterocytes [3], ApoA-IV is synthesized and packaged with lipids and other apolipoproteins into chylomicrons and secreted through the lymphatic system into the blood. Once there, as a member of exchangeable apolipoproteins, ApoA-IV dissociates from chylomicrons and transfers to HDL (high density lipoprotein) particles or circulates in lipid-free form [4, 5].

The past several decades have witnessed numerous advances in identifying physiological functions of ApoA-IV. For instance, ApoA-IV may protect against atherosclerosis [6], mediate reverse cholesterol transport [7, 8], act as an anti-inflammatory agent [9] and modulate intestinal lipid absorption [10]. However, little of these roles were unique to ApoA-IV or not shared by other members of the apolipoprotein family. These led to the creation of ApoA-IV knockout (KO) mice generated by gene targeting technique in 1997 [2]. The most striking phenotype of ApoA-IV KO mice was a reduction in plasma cholesterol and triglyceride levels [2]. And subsequent studies on these KO mice showed more attractive functions of ApoA-IV, including that mediating chylomicron metabolism [11], modulating hepatic TG secretion [12] and improving glucose homeostasis [13].

However, the expression of the neighboring *Apoc3* gene that located in the same gene cluster (*Apoa1*/*Apoc3*/*Apoa4*) with *Apoa4*, was also markedly reduced due to the ApoA-IV KO. As a result, it is hard to determine whether the phenotypes of ApoA-IV KO mice are due solely to the absence of ApoA-IV or may also be related to the deficiency of ApoC-III. Furthermore, the accompanying decrease in ApoC-III expression in ApoA-IV KO mice should never be ignored. To investigate the exact roles of ApoA-IV, TALEN approach could be applied for generating a precise ApoA-IV knockout animal model. TALENs (transcription activator-like effector nucleases) are a widely applicable genome editing approach that have been used to introduce targeted genetic alterations in many model organisms [14, 15]. They comprise an endonuclease catalytic domain tethered to a modular DNA-binding domain that can be easily engineered [14]. Due to their great promise and flexibility for genome editing, TALENs were the preferred gene knockout approach at the time we began our project.

While rats and mice look similar, there are millions of years of evolution separating the rat and mouse [16], and the evolutionary distance between them may be as great as that between humans and Old World monkeys [17]. Moreover, rats are more human-like in many cases of physiology [16]. So using rats may offer better opportunities to uncover the precise roles of ApoA-IV. In the current study, we generated an ApoA-IV knockout rat model by TALEN approach, and then analyzed physiological properties and energy homeostasis of these ApoA-IV knockout rats. In addition, we also performed RNA-seq analysis in liver and inguinal white adipose tissue (iWAT) of rats at various ages. These data collectively suggest that ApoA-IV involves in the regulation of glucose and lipid metabolism of rats.

## Methods

### Rats

*Apoa4*^+/−^ Sprague-Dawlay rats were generated using TALEN approach by Beijing Biocytogen Co., Ltd. (Beijing, China). Homozygous knockout rats and their wild-type (WT) littermates were acquired using heterozygous mating pairs. The genotypes were confirmed by PCR analysis followed by Sanger sequencing and agarose electrophoresis (PCR forward primer: 5′-TATCCCAACTCCAACATCATCCA-3′, reverse primer: 5′-TCGCAGTCTGATCCCACTTACTT-3′). The knockout allele generated a band at 237 bp, while the wild-type allele was at 245 bp. All rats were housed in a temperature-controlled specific pathogen-free (SPF) barrier system, and maintained on a 12-h light/dark cycle (lights on at 7 a.m.). All experiments were carried out on 8–16 weeks age-matched males unless otherwise indicated. Experimental rats were given *ad libitum* access to normal chow diet and sterile tap water, and were gently handled several times every week after weaning to reduce potential stress during tests. All rat experiments were performed in strict accordance with protocols and procedures approved by the Institutional Animal Care and Use Committee of Shanghai Institute of Biochemistry and Cell Biology, Chinese Academy of Sciences.

### Body weight and food intake

Body weights were measured every 2–3 days from weaning until 8 weeks of age and were then monitored weekly. For body fat measurement, rats were anaesthetized with sodium pentobarbital and rapidly dissected. Then visceral white adipose tissue (including epididymal/gonadal, retroperitoneal, perirenal and mesenteric WAT), inguinal subcutaneous WAT and interscapular brown adipose tissue were carefully isolated, cleaned of unrelated materials and weighed.

For food intake measurement, rats were first acclimated to individual housing for at least 1 week. Food were then weighed before administration to each rat, and were weighed again after 24 h to determine absolute daily food intake. The measurements lasted for at least 1 week. To assess feeding behavior following an overnight fast, rats were deprived of food for 16 h (starting at 5 p.m.) while given free access to water. Food intake was then monitored continuously at indicated time points.

### Blood metabolic parameters measurements

Blood metabolic parameters were detected under different physiological conditions, *i.e.*, random-fed state and 16 h fasted state. Whole-blood glucose levels were determined using a hand-held glucose monitor (ACCU-CHEK, Roche Diagnostics), and blood was obtained from the tail vein. For serum preparation, the blood was collected into sterile 1.5 mL tubes and was allowed to clot by standing undisturbed at room temperature for 1 h. The samples were centrifuged at 2000×g for 10 min at 4 °C and the resulting supernatant was immediately apportioned into aliquots and stored at − 80 °C. For plasma preparation, the blood was collected into EDTA-treated tubes (BD Vacutainer) and mixed gently. Blood cells were removed by centrifugation at 2000×g for 10 min at 4 °C and the resulting supernatant was immediately apportioned into aliquots and stored at − 80 °C. Serum insulin was measured with Rat Insulin ELISA kit (Mercodia, Sweden). Serum triglyceride and cholesterol levels were measured with LabAssay Triglyceride and LabAssay Cholesterol kits (Wako, Japan), respectively. Serum free fatty acids were measured with a free fatty acid quantification kit (BioVision, USA). Plasma glucagon was measured with Rat Glucagon ELISA Kit (Wako, Japan). All measurements were in accordance with the manufacturer’s instructions.

### Glucose tolerance, insulin tolerance and pyruvate tolerance tests

Rats were placed in new cages prior to starvation. For GTTs, 16 h fasted rats were injected intraperitoneally (i.p.) with a bolus of glucose (2 g/kg body weight). For ITTs, 6 h fasted rats were injected i.p. with insulin (1 U/kg body weight). For PTTs, rats were fasted for 16 h and injected i.p. with pyruvate (1.5 g/kg body weight). Then whole-blood glucose levels were measured at indicated time points.

### Oral lipid tolerance tests and i.p. intralipid tolerance tests

Rats were fasted for 16 h and then received an intragastric bolus of olive oil (1 mL/100 g body weight per rat). Blood samples were taken from the tail vein, and serum TG levels were measured before and at 1, 2, 4, and 6 h after gavage. For intralipid tolerance tests, rats were fasted for 16 h and then received a single intraperitoneal injection of Intralipid (20% emulsion obtained from Sigma-Aldrich, 1 mL/100 g body weight per rat). Blood were collected and serum TG levels were measured before and at 2, 4, and 6 h after injection.

### Fast performance liquid chromatography

To determine lipoprotein profiles and lipid distributions, 300 μL of pooled serum from 16 h fasted rats were diluted with 100 μL PBS, and loaded onto a Superose 6 10/300 GL column (GE Biosciences). The samples were then eluted with PBS at a constant flow rate of 0.5 mL/min, and fractions of 500 μL were collected and further assayed for TG and cholesterol.

### Hepatic lipid analysis

Hepatic lipids were extracted using the Folch method [18] with minor modifications. In brief, 16 h fasted rats were sacrificed and the liver median lobe was carefully dissected. 20–30 mg of liver tissue were weighed and homogenized in 200 μL of normal saline on ice. Then 1 mL of chloroform/methanol (vol/vol, 2:1) were added directly to the homogenized sample, vortexed vigorously for 30 min and incubated at room temperature for 10 min. The mixture were centrifuged (400×g for 10 min) to separate into a lower organic phase, an interphase, and an upper aqueous phase. The organic phase was carefully transferred into a new tube and dried under nitrogen. The remained lipids on the side and bottom of the tube were completely dissolved in absolute ethanol. The total TG and cholesterol were determined using kits mentioned above.

### Hepatic very low density lipoprotein secretion assay

The hepatic TG secretion was assayed as described previously [19]. Rats were fasted for 16 h and received an i.p. injection of 1 g/kg body weight Poloxamer 407 (P-407, a.k.a. Pluronic F127, Sigma-Aldrich), an inhibitor of lipoprotein lipase. Immediately before and 1, 2, 4, 6 h post P-407 administration, tail vein blood were collected. Serum TG and cholesterol levels were measured as described above. Rats were allowed to recover for at least 1 week before performing other experiments, owing to the slow pharmacokinetics of P-407 [20].

### Western blot analysis

For serum apolipoprotein measurements, 1 μL of serum was combined with 100 μL of loading buffer and heated at 95 °C for 10 min. Then 10 μL of the mixture were separated on SDS-PAGE gels. For tissues or cells, samples were lysed in a Cell Lysis Buffer for Western and IP (Beyotime, China) according to the manufacturer’s instructions. The lysates mixed with loading buffer were heated at 95 °C for 10 min and separated on SDS-PAGE gels. The separated proteins were transferred to PVDF membranes (Immobilon-P, Millipore, USA), and blocked for 1 h at room temperature with 5% non-fat dry milk dissolved in TBS-T (Tris buffered saline with 0.05% Tween-20). The membranes were then probed with indicated antibodies and visualized by LAS4000 (Fujifilm, Japan).

Primary antibodies were as follows: ApoA-IV (Santa Cruz Biotechnology, sc-19040, sc-19036), β-actin (Santa Cruz Biotechnology, sc-1616), α-tubulin (Sigma, T6199), L-PK (Proteintech, 22456–1-AP), GCK (ABclonal, A6293), FASN (ABclonal, A6273). Secondary antibodies were conjugated to horseradish peroxidase and purchased from Santa Cruz Biotechnology.

### RNA extraction and quantitative real-time PCR

Total RNA from tissues was isolated using TRIzol Reagent (Invitrogen, USA). 1 μg of total RNA was reverse-transcribed to cDNA using a ReverTra Ace qPCR RT Kit (TOYOBO, Japan). The cDNA samples were then applied to quantitative real-time PCR (Q-PCR) amplification. Q-PCR reactions were performed with SYBR Green (TOYOBO, Japan) using a Bio-Rad CFX96 Real-Time PCR system. Relative mRNA expression levels were calculated by the ΔΔC_T_ method using *Actb* as an internal control. All primers used for Q-PCR were listed as follows:

- *Actb*: 5′-AAGTCCCTCACCCTCCCAAAAG-3′ and 5′-AAGCAATGCTGTCACCTTCCC-3′.
- *Apoa4*: 5′-GCCAATGTGATGTGGGACTA-3′ and 5′-TTTGTCCTGGAAGAGGGTATTG-3′.
- *Apoc3*: 5′-GCAGGAGTCTGATATAGCTGTG-3′ and 5′-CCAGAGGCCAGTGAACTTATC-3′.
- *Gck*: 5′-ATGCTGGTCAAAGTGGGAG-3′ and 5′-TGTCAAGGAAGTCAGAGATGC-3′.
- *Pklr*: 5′-GACCCGAAGTTCCAGACAAGG-3′ and 5′-ATGAGCCCGTCGTCAATGTAG-3′.
- *Acaca*: 5′-TGTAGAAACCCGAACCGTGG-3′ and 5′-CTGGAAACCAAACTTGGCCG-3′.
- *Fasn*: 5′-ACCTCATCACTAGAAGCCACCAG-3′ and 5′-GTGGTACTTGGCCTTGGGTTTA-3′.
- *Scd1*: 5′-GCCCCTACGACAAGAACATT-3′ and 5′-TGGTGAAGTTGATGTGCCAG-3′.
- *Srebp1*: 5′-CTGTCGTCTACCATAAGCTGCAC-3′ and 5′-ATAGCATCTCCTGCACACTCAGC-3′.
- *Mlxipl*: 5′-CGTGTCCGTGCATTTTACCC-3′ and 5′-TCTCTGCTTTGGGGACAACC-3′.
- *Hmgcr*: 5′-GCCTCGACCTAATGAAGAGTG-3′ and 5′-AGTTTGTAGGCTGGGATGTG-3′.
- *Pgc1*: 5′-ATGGATATACTTTACGCAGGTCG-3′ and 5′-TGGAAGCAGGGTCAAAATCG-3′.
- *G6pc*: 5′-GGCTCACTTTCCCCATCAGG-3′ and 5′-ATCCAAGTGCGAAACCAAACAG-3′.
- *Pck1*: 5′-CAGGAAGTGAGGAAGTTTGTGG-3′ and 5′-ATGACACCCTCCTCCTGCAT-3′.

### RNA-seq and differentially expressed genes analysis

RNA samples were derived from 9 WT and 9 KO rats at three developmental stages–that is adult (18 weeks old), mild-aged (45 weeks old) and aged (90 weeks old). For each rat, liver and inguinal WAT tissues were evaluated (*n* = 3 WT, 3 KO). All samples were flash-frozen in liquid nitrogen and store at − 80 °C until RNA-seq analysis. All subsequent procedures including RNA isolation, library construction and sequencing were executed by Shanghai Biotechnology Corporation (SBC, Shanghai, China). Briefly, total RNA was isolated using RNAiso Plus (Takara, Tokyo, Japan) according to the manufacturer’s instructions. RNA integrity was evaluated by a RIN number using an Agilent Bioanalyzer 2100 (Agilent Technologies, Santa Clara, CA, USA). Qualified total RNA was subjected to further purification using RNAClean XP Kit (Beckman Coulter, CA, USA) and RNase-Free DNase Set (QIAGEN, Germany). Highly abundant rRNAs were removed by poly (A) selection and the obtained mRNA was purified, fragmented, used to synthesize first- and second-strand cDNA followed by end repair and adenylation of 3′ ends. Unique adapters were then ligated to the samples followed by PCR amplification. RNA-seq libraries were quantified using Qubit 2.0 Fluorometer (Life Technologies, USA) and qualified using Agilent Bioanalyzer 2100. Cluster was generated by cBot according to cBot User Guide and sequenced on the Illumina HiSeq 2500 (Illumina, USA) according to Illumina User Guide.

Sequencing raw reads were filtered by removing adapter sequences, short-fragment reads, rRNA reads and other low quality reads. The cleaned high quality reads were mapped to rat genome (Rnor6, Ensembl) using Hisat2 (version 2.0.4). Fragments mapped to the genome were quantified using Stringtie (version 1.3.0) and normalized using TMM (trimmed mean of M) values. FPKM (fragments per kilobase of exon model per million mapped reads) was then calculated by Perl script and used for differentially expressed genes analysis by edge R package. The genes with false discovery rate (FDR) adjusted *p* values < 0.05 and fold change (FC) ≥ 2 (or ≤ 0.5) were considered to be differentially expressed.

### Statistics

Data are presented as mean ± S.E.M. All results were analyzed with OriginPro9.0 software (OriginLab). Unpaired two-tailed Student’s *t* test was used for comparison between two groups. Differences were considered significant at *p* value less than 0.05.

## Results

### Establishment of ApoA-IV KO rats

**Fig. 1:**
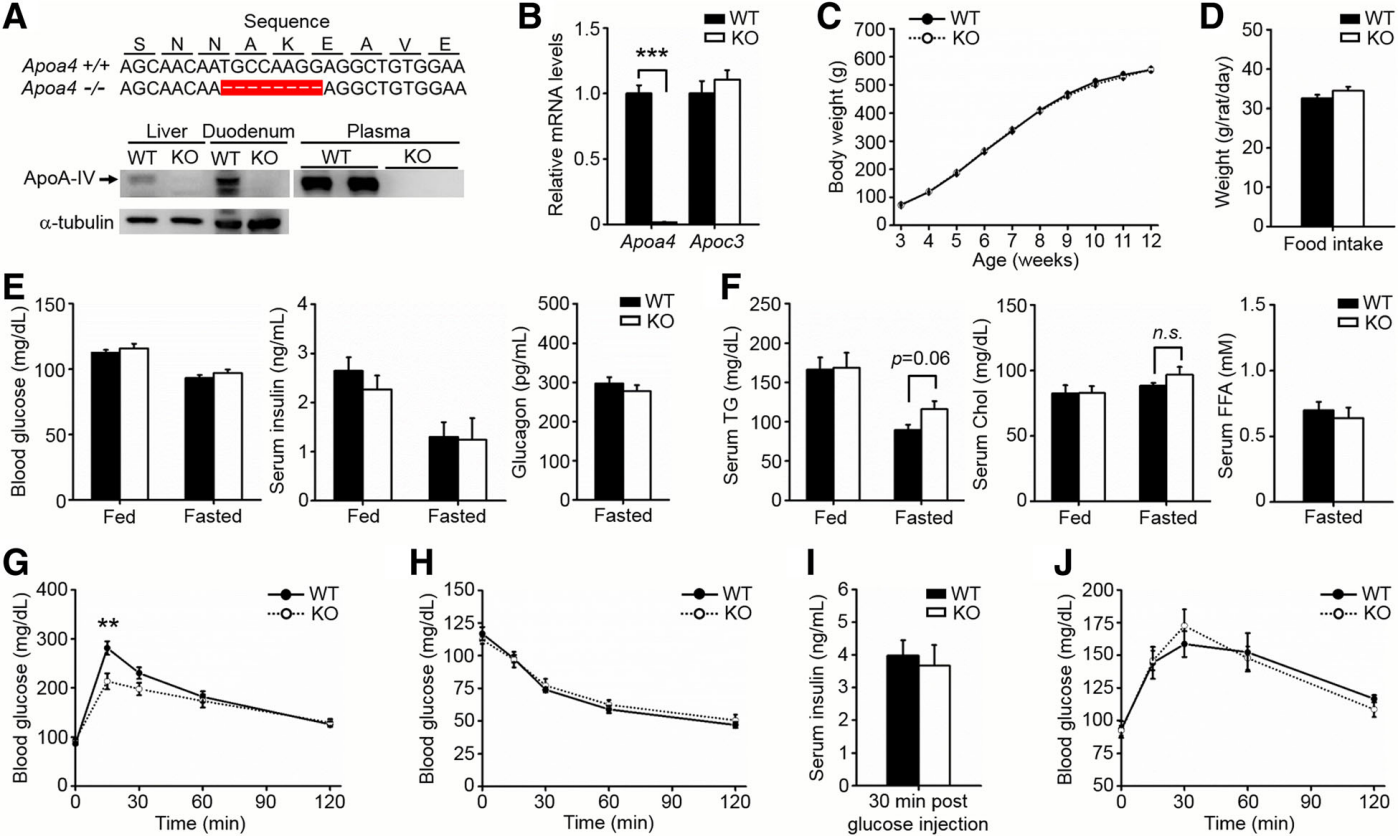
Construction and functional analysis of ApoA-IV knockout rats. **a** Deletion of 8 bp within the *Apoa4* exon 2 using TALEN approach causes a frameshift, resulting in gene knockout. Western blot analysis confirmed deletion of ApoA-IV in the duodenum, liver and plasma. **b** Hepatic mRNA levels of *Apoa4* and *Apoc3* were detected. *n* = 6 WT, 4 KO. **c** Body weight of WT or KO rats when maintained on a normal rodent diet. *n* = 13 WT, 15 KO. **d** Food intake. *n* = 7 WT, 9 KO. **e** Blood glucose (*n* = 10), serum insulin (*n* = 4) and plasma glucagon (*n* = 4) levels were measured under random fed and 16-h fasted states. **f** Serum triglyceride (TG), cholesterol (Chol) and free fatty acid (FFA) were measured under random fed and 16-h fasted states. *n* = 6. **g** IPGTT (2 g/kg body weight). *n* = 6. **h** IPITT (1 U/kg body weight). *n* = 6. **i** Serum insulin levels after glucose injection. *n* = 4 WT, 5 KO. **j** IPPTT (1.5 g/kg body weight). *n* = 4 WT, 5 KO. **p < 0.01, ***p < 0.001 for KO versus WT, n.s., not significant. Error bars indicate S.E.M.

Considering the inaccuracy of existing ApoA-IV KO mice in investigating the physiological roles of ApoA-IV, we generated ApoA-IV specific knockout rats using TALEN approach. As shown in Fig. 1a, an 8 bp deletion was induced within the coding region of *Apoa4* gene, which results in a frameshift and gene knockout. As expected, ApoA-IV expression was greatly diminished in the duodenum and liver of KO rats, and was almost undetectable in the plasma (Fig. 1a). Moreover, the hepatic *Apoa4* mRNA levels were also efficiently decreased in KO rats, while *Apoc3* mRNA levels remained unchanged (Fig. 1b).

### Functional analysis of ApoA-IV KO rats

To determine the functional significance of ApoA-IV ablation, age-matched KO rats and their WT littermate controls maintained on a standard rodent diet were studied. No differences were observed in body weight and body fat mass between KO and WT groups (Fig. 1c, Additional file 1: Figure S1a). Previous reports indicated ApoA-IV might serve as a satiety factor and reduce food intake [21, 22]. However, we did not find the significant physiological differences between KO and WT rats in long-term food intake over the course of several weeks and short-term food consumption following an overnight fast (Fig. 1d, Additional file 1: Figure S1b). Furthermore, the metabolic parameters, including blood glucose, insulin, glucagon, triglycerides (TGs), cholesterol (chol) and free fatty acids (FFAs) were also indistinguishable among these two groups, regardless of starvation or *ad libitum* feeding (Fig. 1e and f). It is worth noting that the fasting serum TG levels in KO rats were slightly increased, which is contrasted with the hypolipidemia phenotype of KO mice [2, 11]. We also screened many more serum parameters using a biochemical analyzer, but failed to find any intriguing differences between the two genotypes (Additional file 1: Figure S1c).

### ApoA-IV KO rats have improved glucose tolerance

When challenged with an intraperitoneal injection of glucose, ApoA-IV KO rats showed rapid glucose clearance, characterized by significantly lowered glucose levels than WT controls 15 min post glucose administration, indicating improved glucose tolerance (Fig. 1g). However, in response to insulin injection, ApoA-IV KO rats exhibited a similar reduction in blood glucose levels, suggesting normal insulin action (Fig. 1h). Since improved glucose tolerance may also be associated with enhanced insulin secretion, we further measured the insulin levels after glucose injection. As shown in Fig. 1i, the insulin concentrations of ApoA-IV KO rats were similar to that of the WT rats at 30 min after glucose injection, which indicates that both groups of the rats have equal ability of insulin secretion. Additionally, the pyruvate tolerance test was performed to estimate the hepatic gluconeogenesis, and the results revealed comparable capacity of ApoA-IV KO and WT rats in converting pyruvate into glucose (Fig. 1j).

Taken together, these results show that ApoA-IV KO rats, compared with the WT rats, display enhanced glucose tolerance but normal insulin secretion ability.

### ApoA-IV KO rats suffer more pronounced fasting-induced hepatic steatosis

**Fig. 2:**
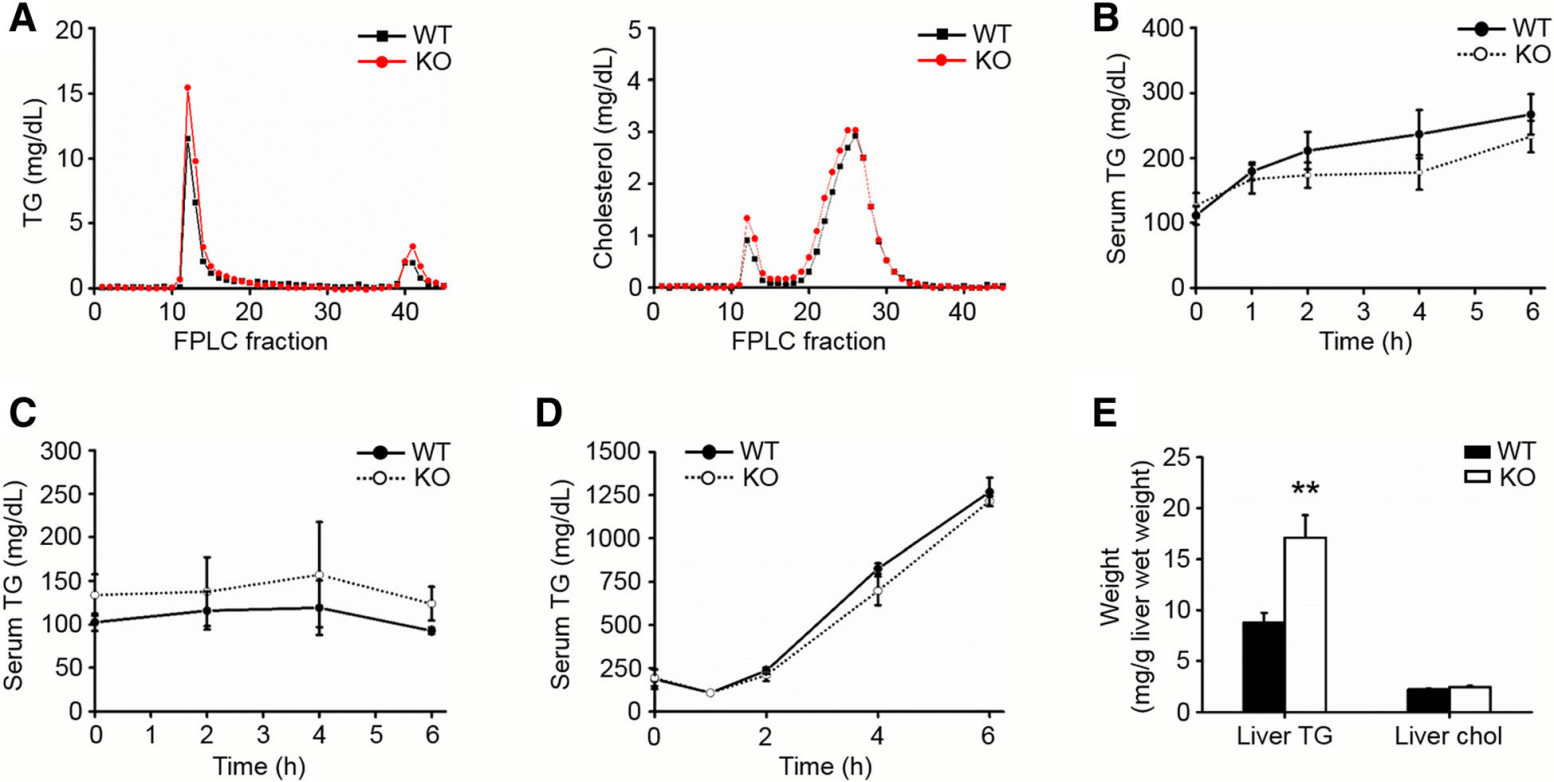
ApoA-IV KO rats have generally normal lipid metabolism but suffer more severe fasting-induced liver steatosis. **a** FPLC profiles of pooled serum from 16-h fasted WT and KO rats. *n* = 5. **b** Oral olive oil tolerance test (n = 5 WT, 4 KO). **c** Intraperitoneal intralipid tolerance test (*n* = 6). **d** ApoA-IV KO and WT rats were fasted for 16-h and treated with Pluronic F-127 to inhibit peripheral lipolysis. Serum TG and cholesterol were measured at 1, 2, 4, 6 h post F-127 injection. *n* = 5 WT, 8 KO. **e** Fasting-induced liver steatosis was more severe in ApoA-IV KO rats. *n* = 7. **p < 0.01 for KO versus WT. Error bars indicate S.E.M.

Since ApoA-IV deficiency tended to increase total serum TG levels, we determine whether the lipoprotein profiles were altered. Serum lipoproteins of 16 h fasted rats were separated by fast performance liquid chromatography (FPLC) followed by analysis of TG and cholesterol. The result showed a mild increase in the VLDL-TG levels in ApoA-IV KO rats compared with that in WT controls (Fig. 2a).

In order to test the ability of postprandial TG clearance, we performed an oral lipid tolerance test with olive oil in 16 h fasted KO and WT rats. Even though the KO rats exhibited a relatively slow increase in serum TG levels, this difference was not significant (Fig. 2b). To bypass the intestinal absorption, we further applied an i.p. intralipid tolerance test to 16 h fasted rats. Unexpectedly, both KO and WT rats showed a marginal increase in serum TG levels that quickly returned to baseline (Fig. 2c). Taken together, these data suggest that ApoA-IV plays minimal role in rat-serum TG clearance under described conditions.

We further addressed whether hepatic TG metabolism was altered in ApoA-IV KO rats. We performed the hepatic TG secretion study, and found that ApoA-IV deficiency did not affect the hepatic TG secretion (Fig. 2d). In addition, the TG and cholesterol levels in the liver were measured. Interestingly, we found that the hepatic TG levels were significantly elevated in ApoA-IV KO rats while the cholesterol levels remained largely unchanged (Fig. 2e). These results indicate that ApoA-IV KO rats suffer a more pronounced fasting-induced hepatic steatosis than WT controls.

### ApoA-IV ablation alters genes expression involved in glycolysis, gluconeogenesis and *de novo* lipogenesis

**Fig. 3:**
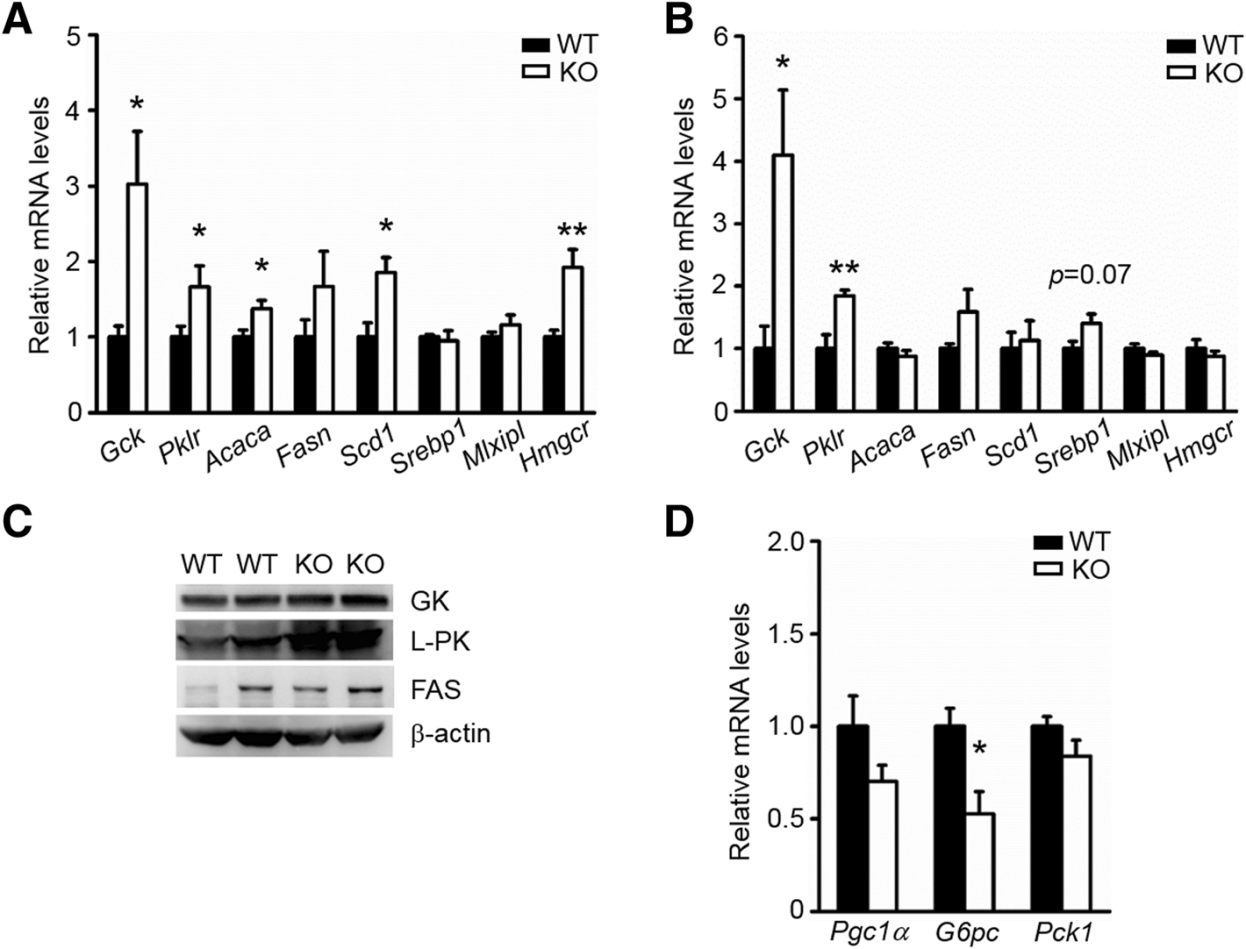
Specific genes involved in glycolysis, gluconeogenesis and *de novo* lipogenesis were altered in ApoA-IV KO rats. **a** mRNA expression levels under fed state. *n* = 6. **b** mRNA expression levels following an overnight fast. *n* = 6. **c** Protein expression levels under fasted state. **d** Gluconeogenic genes were mildly reduced in 16-h fasted KO livers. *p < 0.05, **p < 0.01. Error bars indicate S.E.M.

Considering that the systemic response to insulin was normal in ApoA-IV KO rats (Fig. 1h), we postulated that genes involved in glycolysis and gluconeogenesis may be altered in the absence of ApoA-IV. Thus, we first measured the expression of critical glycolytic genes in liver tissues, including *Gck* (encoding glucokinase) and *Pklr* (encoding pyruvate kinase), and found that both genes were markedly upregulated in random-fed ApoA-IV KO rats compared to WT controls (Fig. 3a). Furthermore, the differences between the KO and WT rats persisted after an overnight fast (Fig. 3b). And there was also an accompanying increase in the protein levels of glucokinase (GK) and liver-type pyruvate kinase (L-PK) in the fasted KO rats (Fig. 3c). We then examined the expression of key gluconeogenic factors, including peroxisome proliferator-activated receptor-γ coactivator 1α (PGC-1α), glucose-6-phosphatase (G6Pase) and phosphoenolpyruvate carboxykinase (PEPCK) in 16 h fasted liver samples. Although all of these factors were downregulated upon ApoA-IV deficiency, only the reduction of *G6pc* mRNA reached statistical significance (Fig. 3d). These data collectively suggest that ApoA-IV deficiency increases glycolysis and attenuates gluconeogenesis, which presumably contribute to improved glucose tolerance.

In addition, we also examined the expression of several critical lipogenic genes, including *Acaca* (encoding acetyl CoA carboxylase), *Fasn* (encoding fatty acid synthase), *Scd1* (encoding Acyl-CoA desaturase), *Srebp1* (encoding sterol regulatory element-binding protein 1), *Mlxipl* (encoding carbohydrate response element binding protein) and *Hmgcr* (encoding 3-hydroxy-3-methylglutaryl-CoA reductase). These genes were somewhat upregulated in ApoA-IV deficient livers and showed an expression pattern towards elevated *de novo* lipogenesis (Fig. 3a and b). This lipogenic genes expression pattern along with the increased glycolysis, which may provide more substrates for FFA and TG synthesis, could account for the elevated hepatic TG content observed in ApoA-IV KO rats.

### RNA-seq analysis on liver and white-adipose tissues of rats at various age-stages

**Fig. 4:**
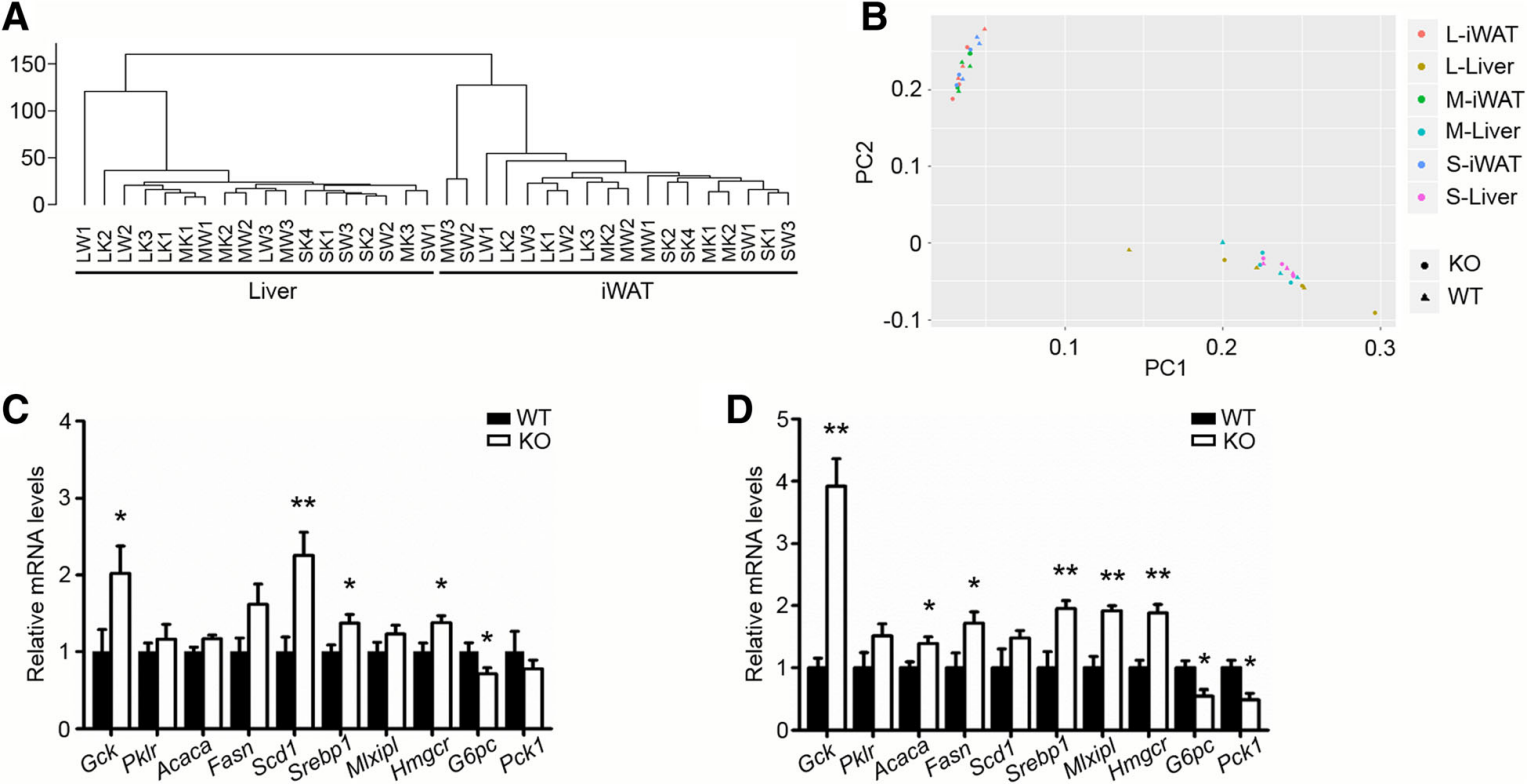
RNA-seq analysis. **a** Hierarchical clustering analysis (HCA). **b** Principal components analysis (PCA), S indicates 18-w, M indicates 45-w, and L indicates 90-w. **c** Gene expression in the liver of 45-w old rats (*n* = 6). **d** Gene expression in the liver of 90-w old rats (*n* = 3).*p < 0.05, **p < 0.01. Error bars indicate S.E.M

To gain a more comprehensive understanding of the involvement of ApoA-IV in glucose and lipid metabolism, we performed RNA-seq analysis on the liver and iWAT tissues of both genotypes. Since previous studies revealed that ApoA-IV levels were significantly and positively correlated with age [23], we selected rats at three developmental stages–that is adult (18 weeks old), mild-aged (45 weeks old) and aged (90 weeks old) for the RNA-seq analysis (3 rats per genotype per age). For each age, differentially expressed genes (DEGs) of liver or iWAT between KO and WT rats were identified based on the FPKM value. The genes with fold change (FC) ≥ 2 (or ≤ 0.5) and *p* values < 0.05 were considered as DEGs. We first performed hierarchical clustering analysis (HCA) and principal components analysis (PCA) to see whether the rats could be well classified into the corresponding groups. As shown in Fig. 4a and b, both HCA and PCA results showed that the samples could be clearly classified into corresponding tissues, but failed to be classified into corresponding genotypes. On the other hand, almost all the DEGs found in the adult rats persisted in mild-aged (45 weeks old) and aged (90 weeks old) rats (Fig. 4c and d), which may indicate that the observed phenotypes in adult rats were stable throughout the life span.

We further evaluated the DEGs of the liver between the adult KO and WT rats in more detail. We found a total of 274 DEGs, including 112 up-regulated and 162 down-regulated genes. And gene functional enrichment analysis was performed using DAVID (version 6.8). The results showed that the up-regulated DEGs were associated with oxygen transport, hydrogen peroxide catabolic process and glucose homeostasis, while the down-regulated DEGs were related to cell migration, inflammatory response and negative regulation of gene expression (Additional file 1: Figure S1d). Moreover, besides the genes mentioned above, other genes responsible for hepatic lipid metabolism, including intracellular lipolysis, β-oxidation, fatty acid esterification and VLDL assembly, were largely unchanged.

Taken together, both Q-PCR and RNA-seq analysis suggest that ApoA-IV deficiency up-regulates genes participated in glycolysis and *de novo* lipogenesis while down-regulates gluconeogenic genes, which results in improved glucose tolerance but more pronounced fasting-induced hepatic steatosis.

## Discussion

ApoA-IV KO mice have long been applied to researches. Previous studies using ApoA-IV KO mice suggested that ApoA-IV improved glucose homeostasis [13] and reduced hepatic gluconeogenesis [24]. However, the present study found that ApoA-IV KO rats had significantly enhanced glucose tolerance, implying that ApoA-IV deficiency improves glucose homeostasis, which is opposite to that of ApoA-IV KO mice. This discrepancy might result from the accompanying deficiency of ApoC-III in ApoA-IV KO mice, which remains to be elucidated. But it is undoubted that ApoA-IV specific knockout rats offer more accurate results when assessing the particular roles of ApoA-IV.

In consistent with an earlier study using ApoA-IV KO mice [11], we also found a significant increase in hepatic TG content in ApoA-IV KO rats. Hepatic steatosis arises from an imbalance between TG acquisition and removal [25]. In the fasted state, hepatic TG were synthesized primarily from FFAs derived from adipose TG hydrolysis and *de novo* lipogenesis [26]. The present study showed that several lipogenic genes were up-regulated in ApoA-IV KO rats, which drives hepatic *de novo* lipogenesis. Moreover, the elevated glycolysis provided more substrates for the usage of lipogenesis, which also contributed to the formation of hepatic TG. Although the previous study on ApoA-IV KO mice indicated that the expression of lipogenic genes were not altered [11], another study showed that the lipogenic genes tended to be up-regulated upon ApoA-IV deficiency [12]. And both studies have investigated the effects of ApoA-IV ablation on hepatic VLDL secretion rate but failed to reach a consensus. We can conclude from these two studies that ApoA-IV deficiency decreases hepatic VLDL size but increases chylomicron size. It seems incompatible because there is an overlap in the particle diameters between chylomicron and VLDL.

The present results of RNA-seq analysis indicate that the expression patterns of the genes mentioned in this study were independent of developmental ages, because the gene expression trends between KO and WT rats were similar among the three age groups (Fig. 3a and b and Fig. 4c and d). But the mechanisms by which ApoA-IV regulates the gene expression remain to be elucidated. In addition, the RNA-seq results showed that there are few significant differences on the expression of genes between KO and WT rats, suggesting that ApoA-IV deficiency has little effect on the regulation of gene expression in general.

## Conclusions

In conclusion, we suggest that ApoA-IV deficiency in rats improves glucose tolerance, attenuates hepatic gluconeogenesis but increases *de novo* lipogenesis in liver. These results may provide a useful reference to the therapeutic approaches to treat type 2 diabetes mellitus and non-alcoholic fatty liver disease.

## Additional file

Additional file 1: **Figure S1. a** Body fat contents. *n* = 7 WT, 5 KO. **b** Food intake after 16-h fasting. *n* = 4*.* **c** Plasma metabolic parameters determined by a biochemical analyzer. *n* = 6. ALT, alanine aminotransferase, U/L; AST, aspartate aminotransferase, U/L; BUN, blood urea nitrogen, mmol/L; CRE, creatinine, μmol/L; HDL-C, high density lipoprotein cholesterol, mmol/L; LDL-C, low density lipoprotein cholesterol, mmol/L; TC, total cholesterol, mmol/L; TG, total triglyceride, mmol/L; LDH, lactate dehydrogenase, U/L; TP, total protein, g/L. **d** Gene Ontology (GO) analysis of up-regulated (left panel) and down-regulated (right panel) genes in livers of rats under random-fed state, enrichment analysis highlighting the top 8 significant biological processes. Error bars indicate S.E.M. (TIF 3087 kb)

## Data Availability

The datasets used and/or analyzed during the current study are available from the corresponding author on reasonable request.

## Abbreviations

ApoA-IV: Apolipoprotein A-IV
ApoC-III: Apolipoprotein C-III
DAVID: The database for annotation, visualization and integrated discovery
DEGs: Differentially expressed genes
FPLC: Fast performance liquid chromatography
GK: Glucokinase
HCA: Hierarchical clustering analysis
HDL: High density lipoprotein
iWAT: Inguinal white adipose tissue
L-PK: Liver-type pyruvate kinase
PCA: Principal components analysis
TALENs: Transcription activator-like effector nucleases
VLDL: Very low density lipoprotein
