# Supplementary figures and images for: Apolipoprotein A-IV involves in glucose and lipid metabolism of rat
Source: Nutr Metab (Lond). 2019 Jul 2;16:41. doi: 10.1186/s12986-019-0367-2 (PMC6604154; doi:10.1186/s12986-019-0367-2)

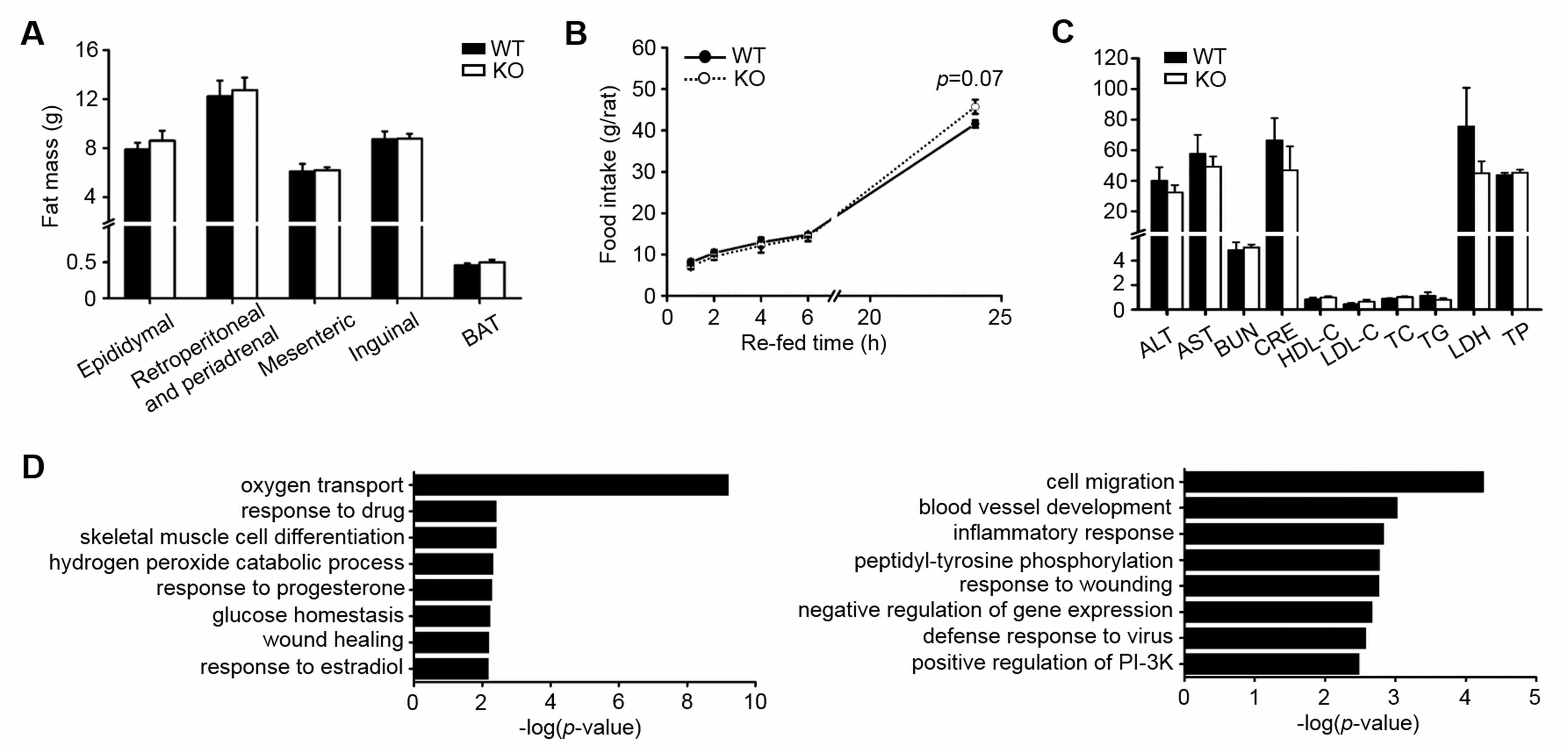

Supplement: Supplementary file 1 — Figure S1. a Body fat contents. n = 7 WT, 5 KO. b Food intake after 16-h fasting. n = 4. c Plasma metabolic parameters determined by a biochemical analyzer. n = 6. ALT, alanine aminotransferase, U/L; AST, aspartate aminotransferase, U/L; BUN, blood urea nitrogen, mmol/L; CRE, creatinine, μmol/L; HDL-C, high density lipoprotein cholesterol, mmol/L; LDL-C, low density lipoprotein cholesterol, mmol/L; TC, total cholesterol, mmol/L; TG, total triglyceride, mmol/L; LDH, lactate dehydrogenase, U/L; TP, total protein, g/L. d Gene Ontology (GO) analysis of up-regulated (left panel) and down-regulated (right panel) genes in livers of rats under random-fed state, enrichment analysis highlighting the top 8 significant biological processes. Error bars indicate S.E.M. (TIF 3087 kb) [file 12986_2019_367_MOESM1_ESM.tif]
